# Management of Moderate‐to‐Highly Exuding Chronic Leg Ulcers With Superabsorbent Wound Dressings Versus Foams Dressings in Polish Settings: An Early‐Stage Cost‐Effectiveness Evaluation

**DOI:** 10.1111/ijn.70147

**Published:** 2026-04-28

**Authors:** Vladica Veličković, Anna Serafin, Beata Mrozikiewicz‐Rakowska, Arkadiusz Jawień

**Affiliations:** ^1^ Evidence Generation Department Hartmann Group Heidenheim Germany; ^2^ Institute of Public Health, Medical Decision Making and HTAUMIT Hall in Tirol Austria; ^3^ Medical Center of Postgraduate Education Warsaw Poland; ^4^ Antoni Jurasz University Hospital No. 1, Collegium Medicum in Bydgoszcz, Nicolaus Copernicus University in Toruń Bydgoszcz Poland

**Keywords:** chronic leg ulcer, chronic wound, cost‐effectiveness, health economics, nurse practitioners

## Abstract

**Introduction:**

Chronic leg ulcers (CLUs) are complex wounds that often fail to heal within a reasonable timeframe, leading to prolonged morbidity, reduced quality of life and significant healthcare costs. The severity and exudate level of these wounds influence treatment choice and patient outcomes. In Poland, evidence on the economic impact of advanced dressings for CLUs remains limited, despite their inclusion in clinical guidelines.

**Aim:**

This study aims to evaluate the cost‐effectiveness and cost‐utility of superabsorbent polymer dressings versus foam dressings in the management of moderate‐to‐highly exuding chronic leg ulcers within Polish healthcare settings.

**Methods:**

This early‐stage model‐based economic evaluation was conducted in a time horizon of 6 months within the context of the Polish healthcare system. The model inputs were identified through systematic literature reviews and incorporated into a mathematical model that accounted for the natural history of chronic leg ulcers.

**Results:**

The average cost per patient for superabsorbent polymer dressings was estimated at PLN 11144 (€2563; $2786), while foam dressings averaged PLN 14685 (€3378; $3671), contributing to the cost difference between the two wound dressings of PLN 3541 (€815; $885) per patient over 6 months. Healing rates are projected to improve by an additional 2%, and health‐related quality of life is expected to increase by 0.123 quality‐adjusted life weeks in favour of superabsorbent polymer dressings.

**Conclusion:**

These results provide evidence that superabsorbent polymer dressings are clinically and economically viable treatment options compared to foams for moderately to excessively exuding chronic leg ulcers in Poland.

## Summary

**What Is Already Known About This Topic?:**

- Superabsorbent polymer (SAP) dressings are a recommended treatment option for management of moderate‐to‐highly exuding chronic leg ulcers (CLUs), which lack economic evaluation in Poland.

**What This Paper Adds?:**

- The cost‐effectiveness analysis predicts that using SAP dressings instead of foam dressings for managing such CLUs could result in a total cost saving of PLN 3541 (€815; $885) per patient over 6 months.
- Over the same period, healing rates are projected to improve by an additional 2%, and health‐related quality of life is expected to increase by 0.123 quality adjusted life weeks (QALWs).

**The Implications of This Paper:**

- This study highlights the potential of SAP dressings as a cost‐saving option for treating moderate‐to‐highly exuding CLUs in Poland.

## 1 Introduction

Chronic leg ulcers (CLUs) are wounds which do not progress through the normal stages of healing in an orderly or timely fashion, often stalling in the inflammatory phase and can take several months or sometimes years, to heal completely (Fukaya et al. 2025; Mosti et al. 2024; National Wound Care Strategy Programme^2023^). Despite the best standard of care currently available, up to 50% of leg ulcers and more than 20% of foot ulcers are not fully healed after 16 and 72 weeks of treatment, respectively (Armstrong et al. 2023; Coye et al. 2025; The 2015). Moreover, these ulcers have a high probability of recurrence after wound healing, more than 50% recurrence (Pennisi et al. 2025). Chronic leg ulcers (CLUs) are a common cause of morbidity, particularly among older adults, and their prevalence varies significantly across countries and populations. A recent systematic review and meta‐analysis estimated the global pooled prevalence of venous leg ulcers (VLUs) at 0.32%, with a pooled incidence of 0.17%, although substantial heterogeneity exists across studies due to differences in methodology and population characteristics (Probst et al. 2023). In Europe, prevalence estimates range from 0.1% to 0.3% in the general population, increasing to over 1% in individuals aged 65 and older (Probst et al. 2023). In Poland, data are limited but suggest a higher burden. A 2012 study reported a prevalence of 1.0% in the general population (Rybak et al. 2012), while more recent estimates from 2016 suggest that up to 1.5% of the population, approximately 600 000 people, were affected (Szumska et al. 2018). The largest Polish epidemiological study, involving 40 095 patients, found active venous ulceration in 0.55% of respondents and either active or healed ulcers in 1.52% (Jawien et al. 2003). Among older adults in Poland, prevalence increases with age, affecting 0.6%–3% of those over 60 and over 5% of those aged 80 and above (Mościcka et al. 2022).

The economic analysis conducted for European countries indicated that the impact of CLUs is considerable. In Germany, the estimated cost per patient for treating a LU is 31.79 PLN (€6.91; $7.46) (Augustin et al. 2014) and in the United Kingdom, with mean costs of 29.10 PLN (€6.31; $6.80; £5.49) per patient (Guest et al. 2020). Regarding the economic burden of chronic wounds for the Polish population, there is a lack of published cost‐of‐illness studies.

The management of CLUs involved a multifaceted approach, considering patient and wound‐related factors, healthcare professional skills and available resources (Atkin 2019). According to current consensus papers and Polish clinical guidelines, SAP dressings are recommended as a first‐line treatment for heavily exuding leg ulcers, and both SAP and foam dressings are recommended for moderately exuding leg ulcers. In addition to these treatments, carboxymethylcellulose dressings and alginates are also recommended (Eriksson et al. 2022; Harding et al. 2019; Jawień Arkadiusz et al. 2011). It is important to note that superabsorbent polymer and foam dressings are typically used as secondary dressings, placed over a primary dressing such as alginates or carboxymethylcellulose. This distinction clarifies that the current analysis focuses on the comparative performance of SAP and foam dressings in their role as secondary dressings for managing moderate‐to‐highly exuding chronic leg ulcers. SAP dressings, known for their high fluid absorbency and compatibility with compression therapy, have emerged as a promising option (Veličković V et al. 2024). Their ability to manage wound exudate, inhibit detrimental metalloprotease activity and provide cushioning while minimizing skin damage made them suitable for highly exuding wounds (Eming et al. 2008; Wiegand and Hipler 2013; Wiegand et al. 2015).

Wound care in Poland is delivered primarily through public outpatient clinics and hospital settings, funded by the National Health Fund (NFZ) (European Observatory on Health and Policies 2024). The system emphasizes compression therapy for venous leg ulcers, supported by dressings such as alginates, hydrocolloids, foams and superabsorbent polymers (SAPs). Access to advanced wound dressings is often limited by reimbursement policies, which differ from countries like Germany or the UK where broader coverage and specialized wound care centres are common (Sopata 2023). In Poland, community nurses play a key role in chronic wound management, but resource constraints and longer waiting times for specialist consultations can impact outcomes. Compared to Western Europe, Poland has fewer dedicated wound care clinics and less integration of multidisciplinary teams, making cost‐effectiveness and practical dressing choices particularly important.

Recent systematic reviews and economic evaluations highlight SAP dressings as a cost‐effective option for heavily exuding wounds, offering improved exudate management and patient comfort compared to foams (V et al. 2024; Veličković et al. 2022). Clinical studies also report that SAP dressings can reduce dressing change frequency and nursing time, which is critical in resource‐limited settings. Given these differences, comparing SAP and foam dressings is clinically relevant for optimizing wound care strategies in chronic leg ulcers.

Given the increasing prevalence and economic burden of CLUs, coupled with the diversity of available dressing types and the lack of economic evaluations for Poland, this study aimed to evaluate the cost‐effectiveness and cost‐utility of SAP dressings versus foam dressings in the management of moderate‐to‐highly exuding CLUs within Polish healthcare settings.

## 2 Material and Methods

The economic evaluation was conducted in line with international guidelines (Briggs et al. 2012; Caro et al. 2012; Eddy et al. 2012; Karnon et al. 2012; Pitman et al. 2012; Roberts et al. 2012; Siebert, Alagoz, Bayoumi, Jahn, et al. 2012), and the Drummond checklist (Drummond et al. 2015) was used to ensure adequate quality of conduct. Quality of reporting followed the CHEERS 2 checklist (Husereau et al. 2022).

### 2.1 Study Design and Setting

This early‐stage model‐based economic evaluation was conducted from the cost perspective of the Polish healthcare system (Narodowy Fundusz Zdrowia, NFZ). Patients treated in outpatient settings were considered the target group for a comprehensive economic evaluation of cost‐effectiveness and cost‐utility between recommended treatment options for managing moderate‐to‐highly exuding CLUs.

### 2.2 Population and Data Sources

The study population comprised patients with CLUs, primarily aged 70 and older, reflecting the demographic most affected by this condition. Data sources for this evaluation included peer‐reviewed literature, existing economic evaluations and clinical trial results relevant to CLU management and SAPs (Table 1) (Atkin et al. 2020; Barrett et al. 2020).

**TABLE 1:** Baseline patient characteristics.

Variable	Mean (observations)	95% CI	
Age	72.79 (78)	69.88	75.69
Gender, males (%)	86.90 (83)	71.55	100
Number of wounds	1 (84)	—	—
Duration of wounds (months)	15.09 (78)	8.34	21.84
Wound size SoC (mm2): baseline	5765 (84)	662	10 868
Wound size SoC (mm2): after 2 weeks	3578 (84)	1923	5233

Abbreviations: CI, confidence interva; observations, number of observationsl.

In this study, chronic CLUs refer specifically to venous leg ulcers and mixed aetiology ulcers (e.g., venous–arterial), which are the most common types managed with compression therapy and advanced dressings. Diabetic foot ulcers were not included in the analysis, as they follow a different clinical pathway and require distinct management strategies.

### 2.3 Treatment Modalities

The intervention used for this evaluation was a superabsorbent dressing (Zetuvit Silicone Border, HARTMANN GROUP), with foam dressings serving as the comparator, as they are the standard of care for managing moderate to heavily exuding CLUs in Polish facilities (Jawień Arkadiusz et al. 2011).

In the model, both groups were assumed to receive standard therapeutic measures recommended for chronic leg ulcer management in Poland, in addition to the evaluated dressings. These measures were modelled as identical across groups to isolate the effect of the dressings. Standard care included wound cleansing with saline or Ringer's solution, application of antiseptics (e.g., polyhexanide, octenidine and povidone‐iodine), skin care and protection products (such as hyaluronic acid gels and vaseline‐based ointments), and compression therapy using compression stockings. Analgesics and systemic antibiotics were incorporated when clinically indicated, following local guidelines.

### 2.4 Overview of Cost Components

The cost analysis involved direct medical costs only. Direct costs included the expenses related to the acquisition of dressings (SAPs and foams), hospital or clinic visits, wound care treatments and any additional medical interventions required for CLU management.

### 2.5 Methods for Cost Estimation

The cost per health state was calculated using the recommended methodology for chronic wounds. A systematic review of cost‐of‐illness studies in Poland did not identify any suitable studies adhering to this methodological approach (Harding, Posnett, and Vowden 2013). Consequently, relevant Polish costs were applied to all resource components of chronic wound care to establish the baseline cost for a static ulcer (UHS3). To determine appropriate costs for other health states, calibration factors that differentiate costs between various health states were derived from the same methodology paper.

The cost of dressings was considered separately, not as part of the health state costs. Dressing sizes were matched to wound sizes for each patient in the model to mimic clinical scenarios. The average cost per dressing type was estimated from a referenced source in 2023 and the frequency of dressing changes per type. All cost data inputs used in the model are specific to Poland, presented in Polish zloty (PLN), with adjustments for inflation and purchasing power parity where applicable (Table 2). No discount rate for costs and outcomes was applied, given the time horizon was less than 1 year.

**TABLE 2:** Cost values for each health state.

Health state	UHS1	UHS2	UHS3	UHS4	UHS5
Cost per health state	PLN 2.90	PLN 42.01	PLN 48.29	PLN 159.45	PLN 637.15

*Note:* djusted for all patient using the risk prediction model.

Abbreviations: D, Death; UHS1, Healed; UHS2, Unhealed Grade 1: progressing; UHS3, Unhealed Grade 1: static; UHS4, Unhealed Grade 1, deteriorating; UHS5, Unhealed Grade 2: severe.

### 2.6 Effectiveness Evaluation

Quality‐adjusted life years were considered, but given the 6‐month time horizon, a utility outcome measure of quality‐adjusted life weeks (QALWs) was employed instead. QALWs were calculated by multiplying 1 week of life gained, based on Polish life tables (age and gender‐specific) (Statistics Poland 2021), by the utility value for that period, as measured in the primary studies. Utility values range from zero (death) to one (perfect health), with inputs informed by published literature. Identification and use of health state utilities follow good practice in outcome research (Brazier et al. 2019). The only identified study on utilities for chronic wounds was by Clegg and Guest (2007), and it was used to inform the model inputs. The healing rate was chosen as the primary effectiveness outcome measure (Table 3 and Supporting Information S1: Section 1 Modelling Interventions Effectiveness).

**TABLE 3:** Utility values for each health state.

Utilities per health state	Value	Source
UHS1	1.000	Clegg et al. (Clegg and Guest 2007)
UHS2	0.730	
UHS3	0.640	
UHS4	0.640	
UHS5	0.610	^a^ Assumption based on Matza et al. (Matza et al. 2019)
D	0	NA

Abbreviations: D, death; NA, non applicable; UHS1, healed; UHS2, unhealed Grade 1: progressing; UHS3, unhealed Grade 1: static; UHS4, unhealed Grade 1: deteriorating; UHS5, unhealed Grade 2: severe.

^a^ Assumed similar to health state utility associated with post‐surgical *Staphylococcus aureus* infections.

**FIGURE 1:**
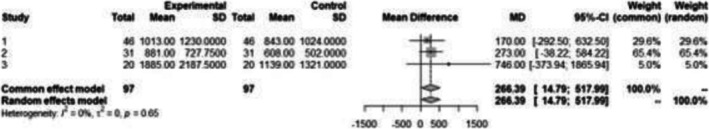
The effectiveness of foam dressings in reducing wound size as measured by the mean difference between baseline and the 14‐day follow‐up.

Baseline characteristics of patients, including age, gender, ulcer duration, number of ulcers, ulcer size, and ulcer grade, were obtained from individual patient data (IPD) in clinical trials to establish baselines for both modelling arms (SAPs and Foams) (Atkin et al. 2020; Barrett et al. 2020). The effectiveness of SAPs was based on the relative ulcer size reduction after 2 weeks of treatment, measured directly from SAP clinical studies (as described in the Population section). The effectiveness of the foam dressing was determined through a systematic literature review and meta‐analysis in moderate‐to‐highly exuding CLUs (complete description in the Supplementary file, 1.1. Foams dressings effectiveness). Foam effectiveness, in terms of relative ulcer size reduction, was applied to baseline patient characteristics from SAP trials to estimate ulcer size reduction after 2 weeks of treatment (Figure 1). Calibration factors for foam effectiveness, conditional on baseline ulcer size, were also informed by the systematic literature review (complete description in the Supplementary file, 1.1. Foams dressings effectiveness).

All patients began in the ‘Unhealed: Grade 1 static’ (UHS3) state and could transition through the rest of the health states according to the transition probabilities depicted in Table 4. The model was developed using MS Excel and Visual Basic for Applications (VBA). Transition probabilities, defined as the likelihood of patients moving from one ulcer health state to another, were informed by previously published benefit‐harm clinical evaluations (Margolis et al. 2004) and risk prediction modelling (Margolis et al. 2004). The transition probabilities from UHS3 to UHS2 (‘Unhealed: Grade 1 progressing’) and UHS3 to UHS1 (‘Healed: skin is intact’) varied over time to reflect changes in pathology based on wound duration, age, gender, number of wounds, wound size, and wound grade. These transition probabilities were predicted using a previously developed risk prediction model that incorporates all the stated characteristics as predictors. Within this model, patients navigate through various health states on a weekly basis. Consequently, we establish transition probabilities to quantify the likelihood of patients moving between health states within a stipulated timeframe. For the purpose of risk quantification, our approach mirrors that of Margolis et al., who utilized logistic regression analysis in their predictive modelling (Margolis et al. 2004). The risk calculation is formalized as follows:

(1) $$P\left(y=1\;\right|x_{j})=\frac{\exp\left(\beta_{0}+x_{\mathrm{age}}\beta_{\mathrm{age}}+x_{\text{gender}}\beta_{\text{gender}}+x_{n_{\text{wounds}}}\beta_{n_{\text{wounds}}}+x_{\text{LnDM}}\beta_{\text{LnDM}}+x_{\text{LnWS}}\beta_{\text{LnWS}}+x_{\mathrm{WG}}\beta_{\mathrm{WG}}\right)}{1+\exp\left(\beta_{0}+x_{\mathrm{age}}\beta_{\mathrm{age}}+x_{\text{gender}}\beta_{\text{gender}}+x_{n_{\text{wounds}}}\beta_{n_{\text{wounds}}}+x_{\text{LnDM}}\beta_{\text{LnDM}}+x_{\text{LnWS}}\beta_{\text{LnWS}}+x_{\mathrm{WG}}\beta_{\mathrm{WG}}\right)}$$

where *p* denotes the probability, *β*_0_ the intercept, *β* the regression coefficient, *x* the independent variable, wounds—number of wounds, *LnDM* logarithm of wound duration in months, *LnWS* logarithm of wound size and *WG* denotes the wound grade.

**TABLE 4:** State‐transition model probabilities.

Transition probability	Value	Source
Transition probability from ‘UHS2’ to ‘UHS1’	0.0250	Panca et al. (Panca et al. 2013)
Transition probability from ‘UHS3’ to ‘UHS2’	Patient specific	Risk‐prediction model Margolis et al. (Margolis et al. 2004) using IPD
Transition probability from ‘UHS3’ to ‘UHS4’	0.0188	Walzer et al. (Walzer et al. 2018)
Transition probability from ‘UHS3’ to ‘UHS1’	0.0188	Meta‐analysis based on Norman et al.
Transition probability from ‘UHS4’ to ‘UHS5’	0.0040	Shannon et al. (Shannon and Nelson 2017)
Transition probability from ‘UHS3’ to ‘UHS5’	0.0170	Walzer et al. (Walzer et al. 2018)
Transition probability from ‘UHS5’ to ‘UHS3’	0.8000	Meta‐analysis based on Norman et al. (Norman et al. 2018)
Transition probability from any health state to death	Age and gender‐specific	Polish life tables

*Note:* Adjusted for all patients using the risk prediction model.

Abbreviations: D, death; IPD, individual patient data; QALWs, quality adjusted life weeks; SAPs, superabsorbent wound dressings; UHS1, healed; UHS2, unhealed Grade 1: progressing; UHS3, unhealed Grade 1: static; UHS4, unhealed Grade 1: deteriorating; UHS5, unhealed Grade 2: severe.

The transition probabilities for the remaining ulcer health states (UHS) were time‐invariant and equal between arms. The model cycle was 1 week to capture important clinical changes in the pace of CLU development.

### 2.7 Model Framework

Consensus papers and Polish clinical guidelines suggest using SAPs as the primary dressing for highly exuding ulcers, despite the lack of robust clinical trial evidence (Erickson et al. 1995; Harding et al. 2019; Jawień Arkadiusz et al. 2011). Thus, this model should be viewed as an early health technology assessment of SAPs, given the preliminary non‐randomized clinical efficacy data used as inputs. In cases where medical decision‐making is uncertain due to limited RCT data, decision‐analytic modelling is advised to support clinicians and payers (Siebert, Alagoz, Bayoumi, et al. 2012).

**FIGURE 2:**
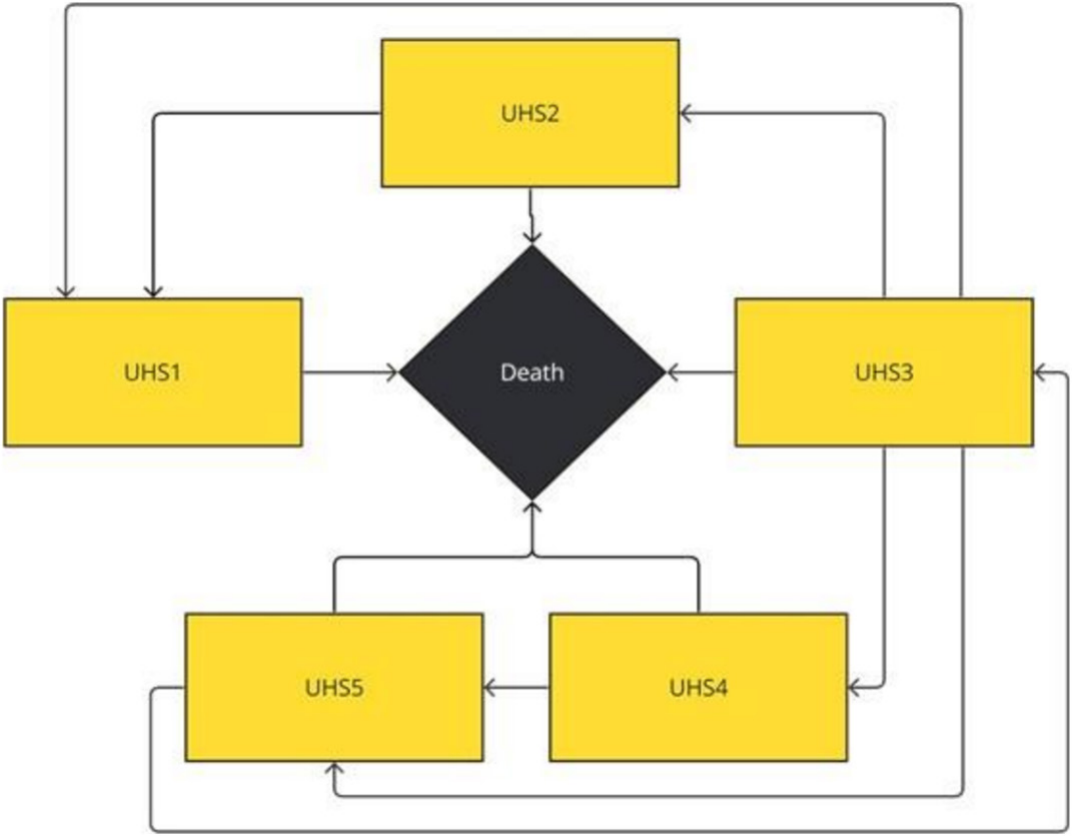
Diagram of the decision analytic model.

An individual patient‐level state‐transition simulation was developed following good modelling practice guidelines (Siebert, Alagoz, Bayoumi, et al. 2012). The model choice was based on the target population's characteristics. Given the heterogeneity of CLUs and the wide range of factors influencing healing rates, microsimulation was deemed the only relevant method to capture all aspects of pathology progression. The model flow and health state structure mimic the natural history of wound healing (Figure 2).

The model uses patient data from clinical studies, assigning baseline characteristics to each simulated patient. Each patient is then duplicated, with one copy assigned to SAP dressings and the other to foam dressings. Patients in both groups traverse health states as shown in Figure 2. After 1000 simulated patients flow through the model for both arms, the final results are averaged across the entire modelled population. Patients transition through the following UHS: ‘Healed: skin is intact’ (UHS1), ‘Unhealed: Grade 1 progressing’ (UHS2), ‘Unhealed: Grade 1 static’ (UHS3), ‘Unhealed: Grade 1 deteriorating’ (UHS4) and ‘Unhealed: Grade 2 severe’ (UHS5). From any health state, a patient can transition to ‘Death’ (Figure 2).

The model's time horizon of 6 months was sufficient to capture the relevant clinical and economic outcomes.

### 2.8 Sensitivity Analyses

To assess the robustness of our cost‐effectiveness results and to account for uncertainty in model parameters, comprehensive sensitivity analyses were conducted in line with good modelling practice (Siebert, Alagoz, Bayoumi, et al. 2012). These analyses explored the impact of varying key input parameters within plausible ranges on the model's outcomes.

Deterministic one‐way sensitivity analysis (OWSA) involved varying one variable's value, which was adjusted to its lower bound (lower confidence interval limit or −20% of the point estimate) and upper bound (upper confidence interval limit or +20% of the point estimate), while keeping all other variables constant to determine its impact on the cost‐effectiveness outcomes (Siebert, Alagoz, Bayoumi, et al. 2012).

In probabilistic sensitivity analysis (PSA) all model parameters were varied simultaneously according to their probability distributions. Appropriate distributions (e.g., beta distribution for probabilities and utilities, gamma distribution for costs) were assigned and Monte Carlo simulations were performed. This provided a more comprehensive view of the uncertainty in the model.

The results of the sensitivity analyses were presented using tornado diagrams for OWSA and scatter plots for PSA. These graphical representations illustrated the parameters that had the most significant influence on the model's outcomes and the probability that SAP dressings were cost‐effective under various willingness‐to‐pay thresholds (WTPs).

## 3 Results

The cost‐effectiveness analysis revealed that the total cost of managing moderate‐to‐highly exuding CLUs with SAP dressings was lower compared to foam dressings. The average cost per patient for SAP dressings was estimated at PLN 11144 (€2563; $2786), while foam dressings averaged PLN 14685 (€3378; $3671). Direct costs, primarily comprising dressing acquisition and wound care treatments, accounted for the majority of these costs. Consequently, the cost difference between the two wound dressings was PLN 3541 (€815; $885) per patient over 6 months (Table 5). The utilization of various resources, their associated cost and other details are presented in Supporting Information S1: Section 2 Resource Use and Cost Analyses.

**TABLE 5:** Cost‐effectiveness evaluation results.

SAPs			Foams			Incremental costs	Incremental QALWs	Incremental HR	ICER (QALY)
Cost	QALWs	HR	Cost	QALWs	HR				
PLN 11144	17.246	34%	PLN 14685	17.123	32%	‐PLN 3541	0.123	2%	‐PLN 28876

Abbreviations: HR, healing rate; ICER, incremental cost‐effectiveness ratio; QALWs, quality adjusted life weeks; SAPs, superabsorbent wound dressings.

Healing rates for SAP‐treated patients were higher, with 34% of ulcers healed within 6 months, compared to 32% in the foam dressing group, representing a 2% higher wound healing rate with SAPs. Patient‐reported outcomes also favoured SAP dressings, with an improvement of 0.123 QALWs (Table 5).

The state‐transition model projected that, over 6 months, patients treated with SAP dressings had a higher probability of complete healing and lower costs. The cost‐effectiveness analysis indicated that SAP dressings were more cost‐effective and dominated foam dressings (a term used in health economics in situations when one technology leads to more favourable outcomes and at the same time leads to lower costs).

### 3.1 Sensitivity Analysis Results

**FIGURE 3:**
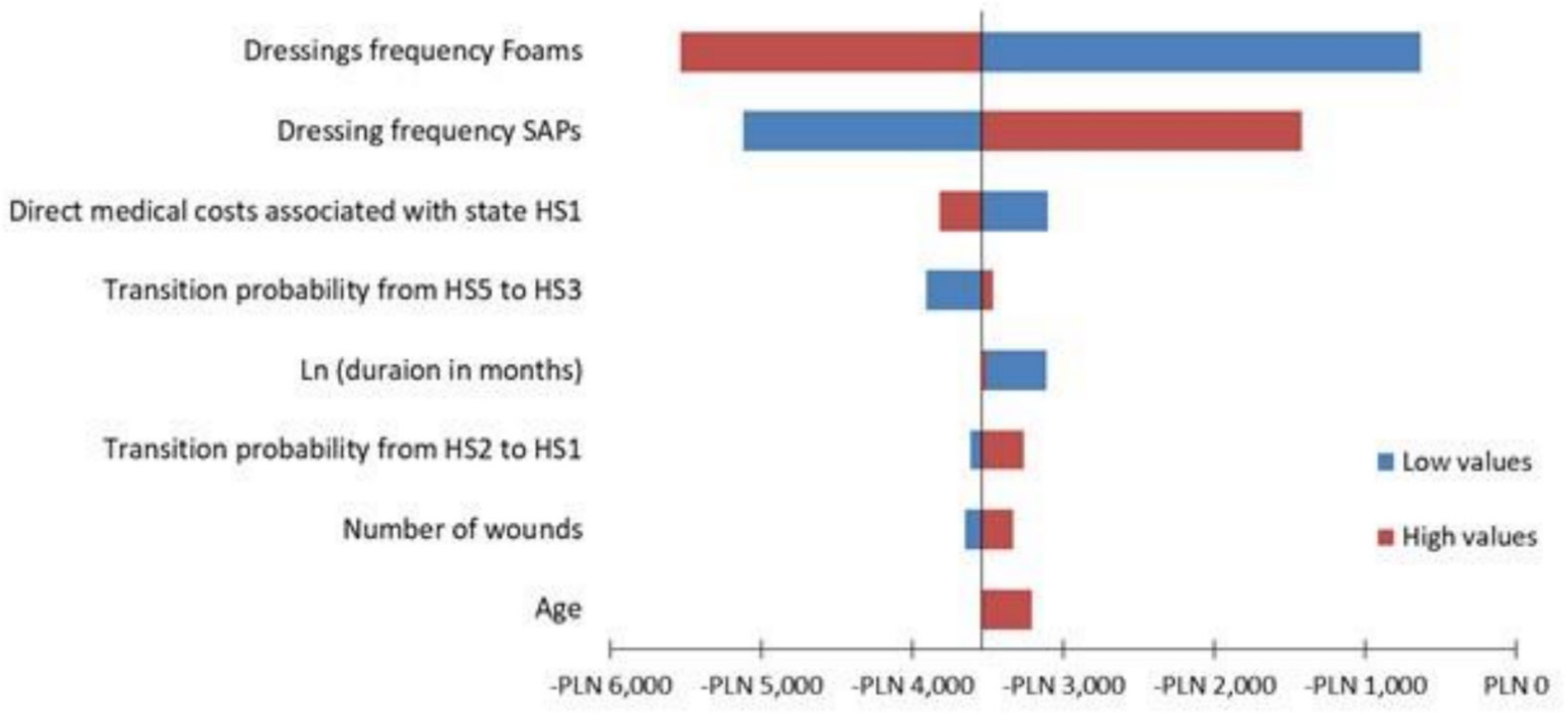
Tornado diagram illustrating one‐way sensitivity analysis.

OWSA indicates that the unit cost of foam and SAP dressings is not the primary driver of overall treatment expenses. Instead, downstream factors, particularly the frequency of dressing changes and the speed of wound healing, play a more decisive role in shaping total costs. Even a seemingly modest improvement of 2% in healing rates can lead to significant reductions in resource utilization and overall care expenditures. These findings underscore the importance of considering clinical performance alongside acquisition costs when evaluating cost‐effectiveness. The manuscript has been updated to reflect these clarifications (Figure 3). However, even in the most impactful variation, cost difference between SAPs and foams never reaches PLN zero or positive; or in other words, in all scenarios, SAPs were cost‐saving.

**FIGURE 4:**
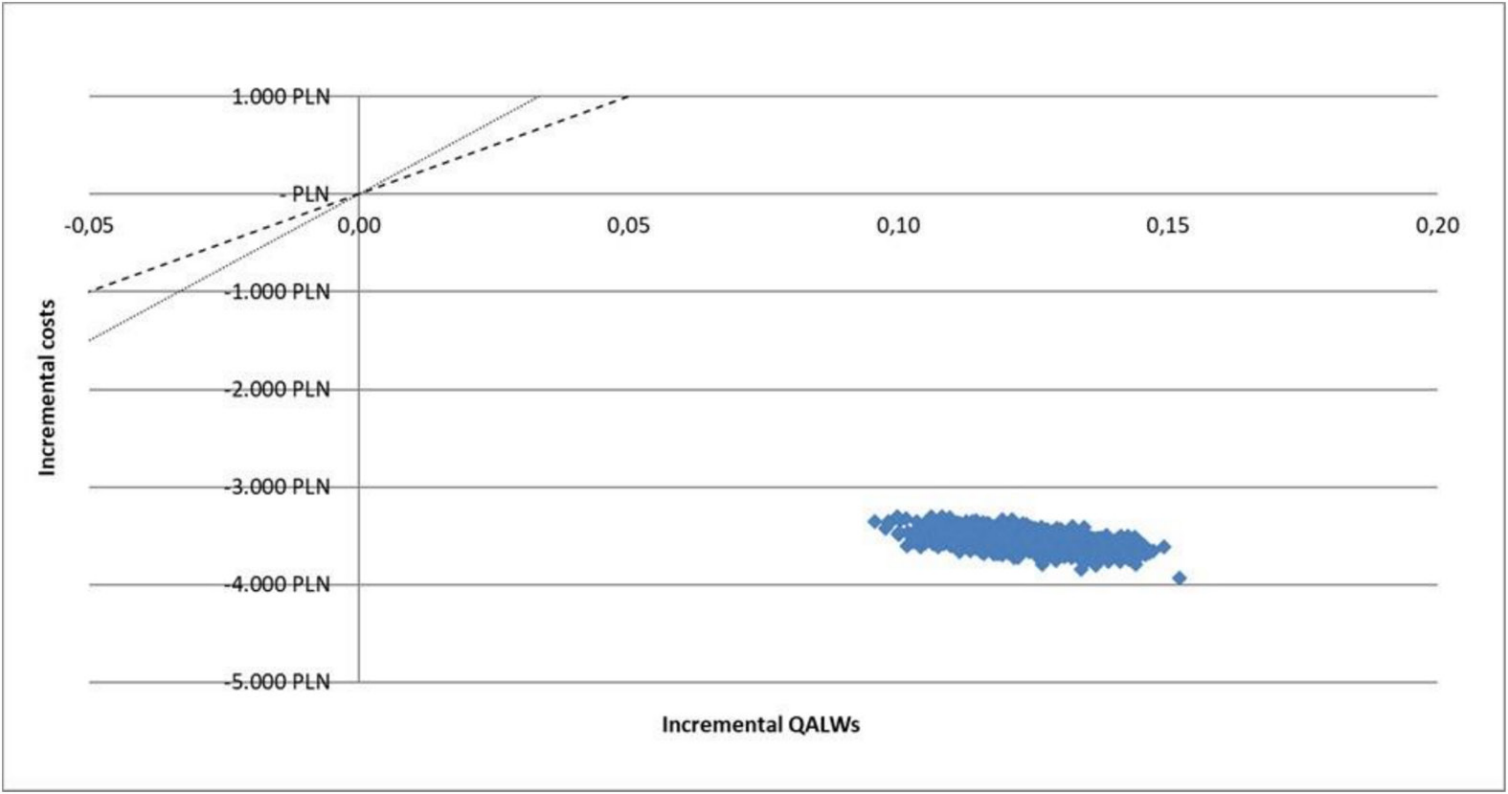
Cost‐effectiveness plane: probabilistic sensitivity analysis results.

PSA demonstrated that SAP dressings remained cost‐saving in 100% of the simulations (Figure 4) at a willingness‐to‐pay threshold of PLN 9000 (€2070; $2250) per QALY (in Poland, the WTP threshold is defined as three times the gross domestic product per capita).

Scenario analyses further supported the robustness of the results, showing that SAP dressings consistently outperformed foam dressings across various clinical and economic scenarios.

## 4 Discussion

The findings of this study highlight the potential of SAP dressings as a cost‐effective solution for managing moderate‐to‐highly exuding CLUs within the Polish healthcare setting. The favourable healing rates associated with SAP dressings not only enhance patient outcomes but also translate into economic benefits. The lower direct costs associated with SAP dressings can alleviate the financial burden on the healthcare system and patients. This is particularly relevant given the increasing prevalence of CLUs in an ageing population and the consequent rise in healthcare expenditures.

Our findings are consistent with those of previous European studies evaluating the cost‐effectiveness profile of SAPs in France, Germany, the UK and Spain (Veličković, Buo, et al. 2026; Veličković et al. 2020; Velickovic, Lembelembe, et al. 2023; Veličković et al. 2022). SAPs are included in clinical guidelines (Evans et al. 2025; Valesky et al. 2024), position papers (Cornelia Wiegand et al. 2015) and consensus documents (Schultz et al. 2019) for the management of moderate‐to‐high exudate chronic wounds. However, our study uniquely contextualizes these benefits within the Polish healthcare system, addressing a gap in the existing literature. The comparative effectiveness of SAP versus foam dressings aligns with international trends in wound care management, emphasizing the need for efficient resource utilization in healthcare (Harding, Apelqvist, et al. 2013; Harding et al. 2013).

The study's results have important implications for clinical practice. The effectiveness of SAP dressings in improving healing rates can lead to a shift in standard wound care practices. Healthcare professionals should consider SAP dressings as a first‐line treatment option for moderate‐to‐highly exuding CLUs, balancing clinical efficacy with cost considerations. Furthermore, the findings can inform policy decisions and guideline updates, advocating for the integration of SAP dressings into national formularies and reimbursement schemes.

While the study provides valuable insights, it is not without limitations. The model‐based approach, though comprehensive, relies on data from existing literature and clinical trials, which may not fully capture the nuances of individual patient experiences (Siebert 2003). Additionally, the 6‐month time horizon, while sufficient for initial healing assessment, does not account for long‐term outcomes and recurrence beyond this period. Transition probabilities were informed by various studies, and it was not always possible to align them perfectly with the same age, comorbidities and medication profiles (Briggs et al. 2012; Eddy et al. 2012; Siebert, Alagoz, Bayoumi, et al. 2012). This is not a limitation specific to this study but rather a general limitation of research on chronic wounds. Due to the lack of data and adequate publications in the literature, distributional effect was not characterized or adjusted to reflect priority populations (Asaria et al. 2015). Although clinicians were involved in the study design and the writing of this paper, the authors did not engage with patients, the general public, communities or payers in the design of the study (Staniszewska et al. 2017).

This analysis did not stratify wounds by classification or severity beyond the inclusion of moderate‐to‐highly exuding chronic leg ulcers. While this was outside the scope of the current economic evaluation, we recognize the importance of understanding cost‐effectiveness across different wound severity levels. Future research should address this aspect to provide more granular insights for clinical and reimbursement decision‐making. Future research should focus on more mature data regarding the comparative clinical effectiveness of dressings. Additionally, more effort is needed in future research to map health‐related quality of life from disease‐specific scales to generic instruments in order to allow for more precise country‐level estimations (Mukuria et al. 2019).

## 5 Conclusion

In conclusion, this study highlights the potential of SAP dressings as a cost‐saving option for treating moderate‐to‐highly exuding CLUs in Poland. Specifically, this cost‐effectiveness analysis predicts that using SAP dressings instead of foam dressings for managing such CLUs could result in a total cost saving of PLN 3541 (€815; $885) per patient over 6 months. Over the same period, healing rates are projected to improve by an additional 2%, and health‐related quality of life is expected to increase by 0.123 QALWs. The findings support the broader adoption of SAP dressings in clinical practice and policy, contributing to improved patient outcomes and sustainable healthcare resource utilization.

## Author Contributions

**Vladica Veličković:** conceptualization (lead), writing – original draft (lead), formal analysis (lead), writing – review and editing (equal). **Anna Serafin:** conceptualization (supporting), writing – review and editing (equal). **Beata Mrozikiewicz‐Rakowska:** review and editing (equal). **Arkadiusz Jawień:** review and editing (equal). All authors critically revised it for important intellectual content, edited and approved its final version.

## Funding

The authors have nothing to report.

## Conflicts of Interest

Vladica Veličković and Anna Serafin are employees of HARTMANN GROUP and may have competing interests. The other two authors declare no conflicts of interest.

## Supporting information


**Table S1:** Transition probability from HS3 to HS1 [^2^].
**Table S2:** Systematic review of foams dressing effectiveness studies (management of moderate‐to‐highly exuding venous leg ulcers only).
**Figure S1:** Foam dressings efficacy in reduction of wound size (mean difference baseline—14 days follow‐up).
**Table S3:** Calibration factors per wound size category.
**Table S4:** Resources utilization and cost for each resource in Poland.
**Table S5:** JPG codes and associated cost per resource use element in Poland.
**Table S6:** Resource use multipliers per health states.
**Table S7:** Cost of dressings.
**Table S8:** CHEERS 2022 Checklist.

## Data Availability

The data that support the findings of this study are available from the corresponding author upon reasonable request.
