# Supplementary material for: Management of Moderate‐to‐Highly Exuding Chronic Leg Ulcers With Superabsorbent Wound Dressings Versus Foams Dressings in Polish Settings: An Early‐Stage Cost‐Effectiveness Evaluation
Source: Int J Nurs Pract. 2026 Apr 28;32:e70147. doi: 10.1111/ijn.70147 (PMC13124319; doi:10.1111/ijn.70147)
Supplement: Supplementary file 1 — Table S1: Transition probability from HS3 to HS1 [2]. Table S2: Systematic review of foams dressing effectiveness studies (management of moderate‐to‐highly exuding venous leg ulcers only). Figure S1: Foam dressings efficacy in reduction of wound size (mean difference baseline—14 days follow‐up). Table S3: Calibration factors per wound size category. Table S4: Resources utilization and cost for each resource in Poland. Table S5: JPG codes and associated cost per resource use element in Poland. Table S6: Resource use multipliers per health states. Table S7: Cost of dressings. Table S8: CHEERS 2022 Checklist. [file IJN-32-e70147-s001.docx]

**Supplementary file**

1. **Modeling interventions effectiveness**

In the context of delineating the natural progression of chronic wound conditions, the adoption of a state-transition modeling, commonly referred to as a Markov model, emerges as the optimal strategy. This methodology affords the flexibility of engaging in either cohort-based or individual-level simulations. Given the heterogeneity inherent in the definition of chronic wounds, characterized by a plethora of etiological subtypes, we have elected to eschew cohort-level analysis in favor of individualized patient simulations. These simulations are tailored to reflect each patient's unique demographic and wound-specific parameters. The technique employed is typically encapsulated under the term 'microsimulation model'. Within this model, patients navigate through various health states on a weekly basis. Consequently, we establish transition probabilities to quantify the likelihood of patients moving between health states within a stipulated timeframe. For the purpose of risk quantification, our approach mirrors that of Margolis et al., who utilized logistic regression analysis in their predictive modeling [^1^]. The risk calculation is formalized as follows:

Eq.1. $P\left( y=1 \right|x_{j})= \frac{exp(\beta_{0}+{x_{age}\beta}_{age}+ {x_{gender}\beta}_{gender}+ {x_{n_{wounds}}\beta}_{n_{wounds}} + {x_{LnDM}\beta}_{LnDM}+{x_{LnWS}\beta}_{LnWS}+{x_{WG}\beta}_{WG})}{1+ exp(\beta_{0}+{x_{age}\beta}_{age}+ {x_{gender}\beta}_{gender}+ {x_{n_{wounds}}\beta}_{n_{wounds}} + {x_{LnDM}\beta}_{LnDM}+{x_{LnWS}\beta}_{LnWS}+{x_{WG}\beta}_{WG})}$

Where p denotes the probability, β_0_ the intercept, β the regression coefficient, x the independent variable, wounds – number of wounds, LnDM logarithm of wound duration in months, LnWS logarithm of wound size, and WG denotes the wound grade.

In our study, patients are conceptualized with initial characteristics that pertain both to the patient and their wounds. These initial factors serve as pivotal predictors within the risk prediction model proposed by Margolis et al [^1^]. Subsequently, each simulated patient is duplicated into two identical entities to examine their clinical outcomes under two distinct treatment scenarios: standard of care (SoC) versus SAPs dressing. Following this bifurcation, the natural progression of each patient's condition is meticulously modeled over a 24-week horizon, employing a sophisticated nested risk prediction model to forecast individual patient trajectories. Upon reaching the conclusion of this period—or prematurely in instances of patient mortality—the cumulative Quality-Adjusted Life Weeks (QALWs) alongside the healing status are comprehensively evaluated. This simulation cycle is methodically replicated for a cohort of 1,000 patients per treatment arm.

Table S1. Transition probability from HS3 to HS1 [^2^]

| **Study** | **Country** | **Size** | **Follow-up** | **Healing %** | **Weeks** | **Rate** | **Risk** |
| --- | --- | --- | --- | --- | --- | --- | --- |
| Fogh, 2012 | Europe | 60 | 1.5 months | 0.1833 | 6.51786 | 0.03106594 | 0.03058835 |
| Gottrup, 2008 | Europe | 60 | 1.5 months | 0.16 | 6.51786 | 0.0267501 | 0.02639548 |
| Harding, 2001 | UK | 65 | 12 weeks | 0.2615 | 12 | 0.02526118 | 0.02494479 |
| Norkus, 2005 | Denmark | 48 | 12 months | 0.4 | 52.14288 | 0.00979665 | 0.00974882 |
| Schulze, 2001 | Germany | 113 | 4 weeks | 0.05 | 4 | 0.01282332 | 0.01274146 |
| Meaume, 2012 | France | 94 | 8 weeks | 0.0745 | 8 | 0.00967764 | 0.00963097 |
| Senet, 2014 | Europe | 94 | 6 weeks | 0.0319 | 6 | 0.00540332 | 0.00538874 |
| Sopata, 2016 | Poland | 50 | 40 weeks | 0.76 | 40 | 0.03567791 | 0.03504895 |
| Vanscheidt, 2012 | Germany | 66 | 12 weeks | 0.32 | 12 | 0.03213854 | 0.03162759 |
| Weighted Average |  |  |  |  |  |  | **0.0187691** |

Table S2. Transition probability from HS3 to HS5 [^2^]

| **Study** | **Country** | **Size** | **Follow-up** | **Healing %** | **Weeks** | **Infection** | **Rate** | **Risk** |
| --- | --- | --- | --- | --- | --- | --- | --- | --- |
| Fogh, 2012 | Europe | 60 | 1.5 months | 0.1833 | 6.51786 | 0.03 | 0.00467319 | 0.00466229 |
| Gottrup, 2008 | Europe | 60 | 1.5 months | 0.16 | 6.51786 | 0.03 | 0.00467319 | 0.00466229 |
| Harding, 2001 | UK | 65 | 12 weeks | 0.2615 | 12 | 0.0833 | 0.00724792 | 0.00722172 |
| Schulze, 2001 | Germany | 113 | 4 weeks | 0.05 | 4 | 0.2 | 0.05578589 | 0.05425839 |
| Meaume, 2012 | France | 94 | 8 weeks | 0.0745 | 8 | 0.0638 | 0.00824077 | 0.00820691 |
| Sopata, 2016 | Poland | 50 | 40 weeks | 0.76 | 40 | 0.12 | 0.00319583 | 0.00319073 |
| Vanscheidt, 2012 | Germany | 66 | 12 weeks | 0.32 | 12 | 0.091 | 0.00795085 | 0.00791932 |
| Weighted Average |  |  |  |  |  |  |  | **0.016956** |

To encompass the inherent uncertainty associated with model inputs, our analysis transitions from a deterministic framework to a stochastic methodology. Herein, we undertake a random sampling process across a plausible spectrum of all decision-analytic model parameters simultaneously. This process is executed adopting a Monte Carlo probabilistic analysis [^3^]. Each iteration is characterized by a unique set of parameters that fall within the predefined distributions' boundaries. The selection of normal, beta, gamma, and Dirichlet distributions for these parameters is guided by established methodological recommendations. The parameters for these distributions are derived using the method of moments, with a standard deviation of 0.5 applied to inputs lacking precise variability measures. This approach not only enhances the robustness of our predictive model by accommodating parameter variability but also underscores the significance of individualized patient trajectories in informing treatment outcomes.

In the development of transition probabilities, we employed a comprehensive methodology utilizing multiple data sources. This process encompassed several critical steps, as outlined below:

Step 1: Initially, relevant probabilities were meticulously identified and extracted from the available data.

Step 2: Initially, probabilities were converted into rates using the formula:$r= - \frac{1}{t}ln(1-p)$, where r is rate and p and t are probability and follow-up time, respectively, as in the original source. Then transformed from rates into the weekly probabilities using equation:

$p=1-e^{-\sum rt}$where r is rate from previous step, t is one week and p probability.

Step 3: To synthesize these weekly probabilities, a weighted average was calculated, employing the sample sizes of the studies as weights to ensure representativeness.

In addition to these methodological steps, rigorous internal model validation was conducted. This involved a standardized quality checklist designed to identify and rectify any technical inaccuracies. Furthermore, an analysis of extremes was performed, evaluating the model's resilience to extreme input values (e.g., life expectancy of zero, utilities at 0 or 1). This comprehensive validation effort, incorporating both extreme value analysis and a detailed quality checklist, to effectively identify and resolve all technical discrepancies within the decision-analytic model.

It is important to acknowledge that our model draws upon the predictive framework established by Margolis et al., thereby necessitating a thorough evaluation of the regression model's technical fidelity as part of our broader validation exercises [^1^]. The predictive model, developed based on a cohort of 20,000 individuals, exhibited an exceptional discrimination value and demonstrated robust validation outcomes. Moreover, logistic regression models have been substantiated as an accurate approach for predicting outcomes over short-term periods, specifically in this instance, spanning 6 months or 24 weeks.

In assessing the results derived from the Margolis et al. risk prediction model, it is imperative to acknowledge and consider its limitations. As delineated in the original description by Margolis et al., several critical issues warrant attention:

- There is variability in the standard therapy administered to patients, indicating that not all individuals received identical treatment regimens.
- The model does not encapsulate all significant predictors, both known and unidentified. This limitation stems from the model's design objective, which was to provide a simplified tool for clinical use, potentially sacrificing comprehensive predictive accuracy for user-friendliness.
- The methodology employed for estimating wound size is not devoid of imperfections, which could impact the model's predictive capabilities.
- Furthermore, it is crucial to recognize that the model was developed based on patient data collected between 1988 and 2000, with its findings published in 2004. Since then, the field of wound care has undergone considerable advancements. Such developments may have significantly altered the landscape in which the model operates, posing a substantial risk that its accuracy at the time of publication has been compromised in the context of contemporary practice.
- This temporal gap underscores the necessity for caution when applying the model's insights to current clinical scenarios, suggesting that its predictions might not fully reflect the present state of wound care advancements.

In health outcomes research, Health-Related Quality of Life (HRQoL) metrics are typically obtained from quality-of-life assessments using generic, non-disease-specific instruments. These metrics are then directly converted into utility values. Alternatively, disease-specific tools may be employed, necessitating the use of a mapping algorithm to convert these disease-specific measures into generic terms, thus facilitating the derivation of utility values. These utility values range from zero (representing death) to one (indicating perfect health), positioning patient HRQoL on a continuum between these two bounds as determined through targeted population surveys.

By integrating utility values with life expectancy data, it is possible to calculate quality-adjusted life weeks (QALWs), enabling an evaluation of the expected QALWs for patients across different treatment arms over a six-month period. In cases where no suitable input data could be identified for specific health states—namely, “Unhealed grade 1: deteriorating (U. G1. D)” and “Unhealed grade 2: severe (U. G2. S)”—assumptions regarding utility values were formulated based on the insights from existing empirical research. For the “U. G1. D” state, utility values were conservatively approximated to match those of the “Unhealed grade 1: static (U. G1. s)” state as reported in the study by Clegg et al [^4^]. Conversely, for the “U. G2. S” state, we referenced the study by Matza et al., which provided utility decrements associated with infections of acute wounds, albeit not chronic ones [^5^]. This approach ensures that the evaluation of health outcomes remains grounded in empirical evidence, albeit with necessary assumptions to address data gaps.

**1.1..Foams dressings effectiveness**

We conducted a search of Embase, Medline, and Cochrane databases for studies that reported the efficacy of foam dressings, using the reduction of wound size after two weeks as an outcome measure in patients with moderate-to-heavily exuding venous leg ulcers. The criteria were chosen to ensure the study population was comparable to that of the superabsorbent polymer (SAP) clinical trials used in this evaluation. Our initial search yielded 5,900 records. After screening titles and abstracts, we selected 25 papers for full-text review. Ultimately, only three studies met our inclusion criteria.

Table S2. Systematic review of foams dressing effectiveness studies (management of moderate-to-highly exuding venous leg ulcers only)

| **Study** | **Age** | **SS** | **Gender** | **Wound duration** | **Wound size baseline** | **WSR**  **(Day 14)** | **Absolute WAR/day** | **Relative WAR/day** |
| --- | --- | --- | --- | --- | --- | --- | --- | --- |
| Vanscheidt et al. ^6^ | 66.40 | 46 | 50% | 10.91 | 1013 | 170 | 12.14 | 1.20% |
| Charles et al.^7^ | 71.00 | 31 | 48.40% | 32 | 881 | 273.11 | 19.51 | 2.21% |
| Szewczyk et al.^8^ | 62.20 | 20 | 40.00% | 17.5 | 1885 | 746.46 | 53.32 | 2.60% |

The meta-analysis incorporated the aforementioned studies to quantify appropriate decision-analytic model inputs for the effectiveness of foam dressings. The results are presented in a forest plot below, using mean difference as the measure of effect. For the meta-analysis, we utilized the R package 'meta.' Given that the data are continuous (mean difference), we employed the 'metacont' function and the built-in summary function 'sm' with Hedges' adjusted g..
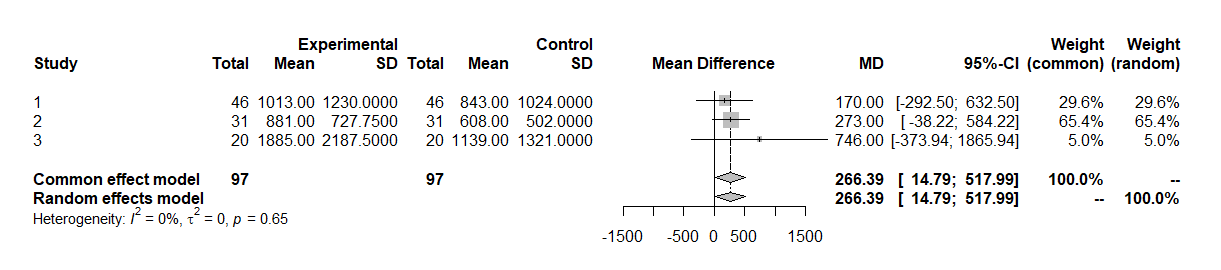


Figure S1. Foam dressings efficacy in reduction of wound size (mean difference baseline – 14 days follow-up).

To ensure the efficacy of foam dressings specific to wound size, calibration factors were estimated based on the study by Szewczyk et al. (2008) ^8^, as reported in the table below.

Table S3. Calibration factors per wound size category

| **Wound size category** | **Calibration factor** |
| --- | --- |
| Wound size category 1 (0-10 cm^2^) | 1.124 |
| Wound size category 2 (10-20 cm^2^) | 0.842 |
| Wound size category 3 (>21 cm^2^) | 1.035 |

1. **Resource use and cost analyses**

Harding et al. introduced an approach in their publication, proposing a comprehensive costing method that provides a valuable framework for health economic modeling in the context of chronic wounds^9^. Their study categorized the natural history of the condition into five distinct groups: Healed (HS1), Progressing (HS2), Static (HS3), Deteriorating (HS4), and Severe (HS5). Each group represents a specific stage of wound progression, ranging from complete healing to severe infection that may require hospitalization or surgical intervention.

To facilitate the economic evaluation of chronic wounds in Poland, a detailed analysis of the resources employed in their treatment was conducted. The utilization of various resources, their associated prices, and the average costs are presented in Table S3 and S4, offering a comprehensive overview of the economic aspects associated with chronic wound management in Poland^10-12^.

According to Harding et al., the main sources of cost for the treatment of HS3 (Static) include dressings (11%), equipment (2.5%), investigations (4.5%), hospital admissions (2.0%), district nurse visits (30.1%), and outpatient clinical visits (47.4%). Therefore, based on this information, the direct medical cost per patient for the static ulcer state in the Polish setting were estimated.

Table S4. Resources utilization and cost for each resource in Poland

| Category | Type | Local recommendation / guideline / year | Typicaly used product | Brand names (examples) | Price per pack (PLN) |  |
| --- | --- | --- | --- | --- | --- | --- |
| Skin care | Topical ointments | 1 | Onion extract + heparin | Contractubex 20 g | 30 |  |
|  |  |  | Chamomille extract+ heparin | Cepan | 15.9 |  |
|  |  |  | Hialuronic acid gel | Hexatiab activ gel 25 ml | 30 |  |
|  | Skin protection | 1 | Vaseline | Vaselinum album 30 ml | 9 |  |
|  |  |  | Linseed oil ointment | Linomag 30 mg | 10 |  |
|  |  |  | Cholesterol ointment | Linocholesterol a+e creme 90 g | 14 |  |
|  | Wound cleansers | 1 | NaCl | 0,9% NACL 500 ml | 7 |  |
|  |  |  | Ringer solution | Płyn Ringera 500 ml | 9 |  |
|  | Antispetics | 1 | Betaine/polyhexanide (PHMB) | Prontosan | 49 |  |
|  |  |  | Octenidine dichloride (OCT) | Linoseptic 30 ml | 9 |  |
|  |  |  |  | MaxiSeptic 50 ml | 19 |  |
|  |  |  |  | Ocenisept 50 ml | 21 |  |
|  |  |  |  | Oktaseptal 60 ml | 14 |  |
|  |  |  | Povidine iodine (PVP-I) [2] | Braunol, 7,5% 250 ml | 60 |  |
|  |  |  |  | Braunovidon®, maść 20 g | 35 |  |
|  |  |  | Hypochlorites | Aqvitox D 250 ml | 52 |  |
|  |  |  |  | Granudacyn 500 ml | 50 |  |
|  |  |  |  | Microdacyn 60® Wound Care | 52 |  |
|  |  | 2 | Silver products | ArgoTiab krem 50 ml | 34 |  |
|  |  |  | Cadexomer iodine | Iodosorb - not available in PL |  |  |
|  | Antibiotic ointments | 1 | *"(…) use of topical antibiotics is considered as ineffective and inducing bacteria resistance increase (…)" "Currently, due to the ineffectiveness of therapy and selection of resistant strains, the use of topical antibiotics is not recommended in the treatment of chronic wounds."* | not applicable | n/a |  |
|  |  | 2 | Metronidazole (solution and gel) | 1% Metronidazol gel Jelfa | 20 |  |
|  | Topical anesthetic ointment | 1 | Licocaine | Emla 5 g | 39 |  |
|  |  |  |  |  |  |  |
|  |  |  |  |  |  |  |
|  |  |  |  |  |  | PLN 27.57 |
| Analgesics and antibiotics for wound | Antibiotics | 3 | Cefalexin | Cefaleksyna TZF 500x16 | 24 |  |
|  |  |  | Amoxicili/clavulonic acid | Augmentin 875+125 14 t | 37 |  |
|  |  |  | Clindamycin | Clindamycin MIP 600 x 16t | 30 |  |
|  |  |  | Levofloxacin | Levofloxacin 500 x 10 t | 25 |  |
|  | Analgesics | 1.3 | Paracetamol | Paracetamol 500 x 20 | 4 |  |
|  |  |  | Ketoprofen | Ketonal 50 mg x 10 t | 9 |  |
|  |  |  | Naproxen | Naxii 220 mg x 10 t | 9 |  |
|  |  |  | Diclofenac | Voltaren 25 mg x 10 t | 11 |  |
|  |  |  | Tramadol | Tramal 50 mg x 20 t | 7.9 |  |
|  | Phelobotropic drugs | 3 | Pentoxiphylline | Pentohexal 600 Retard x 30t | 44 |  |
|  |  |  | Diosmine | Phlebodia 600 x 30 | 29 |  |
|  |  |  | Sulodexide | Vessel duo F x 50 | 70 |  |
|  | Anticoagulants | 1,2,3 | Not included |  |  |  |
|  | Immunosuppressants | 1,2,3 | Not included |  |  |  |
|  | Psychotropics | 1,2,3 | Not included |  |  | PLN 24.99 |
| Compression hosiery | Compression stockings/Lower leg | 3 | Podkolanowki | Veera Przeciwżylakowa | 124.5 |  |
|  | Compression stockings/Upper leg |  | Ponczochy | Veera pończochy | 158 |  |
|  | Compression stockings/Pelvis |  | Rajstopy kompresyjne | Veera rajstopy |  | PLN 141.25 |

Table S5. JPG codes and associated cost per resource use element in Poland

|  | ***Type*** | ***JGP code(s)*** | ***Cost [PLN]*** |
| --- | --- | --- | --- |
| **Investigations (outpatinet)** | Ultrasonography | 88.777 | 42.79 |
|  | Contrast-enhanced MR angiography | 88.978 | 491.26 |
|  | Non-contrast-enhanced MR angiography | 88.977 | 140.63 |
|  | Phlebography | 88.6 | 389.15 |
|  | Biopsy | 86.11 | 42.5 |
|  | Allergy tests | 99.801 | 154.9 |
| **Outpatient clinic visits** | Physiotherapist - consultation | 89.0021 | 20.15 |
|  | Physiotherapist - diagnostic | 93 | 39.95 |
|  | Therapist of lymphatology | 93.3915 | 22.43 |
|  | Nurse - home visit | 89.05 | 90.04 |
|  | Practitioner | 89.02 | 39.95 |
|  | Consultant | 89 | 39.95 |
|  | Specialised outpatient clinic | 23.0105 | 40.24 |
| **Hospital Admission** | Arterial surgery | 39.501 | 2156.38 |
|  | Vein surgery | 38.5 | 998.59 |
|  | Meshgraft transplantation | 86.6 | 809.94 |
|  | Hospitalisation |  | 959 |
|  | Conservative therapy and VAC |  | 701.6 |

Based on the estimated cost for the Static health state, the costs for other health states are extrapolated using multipliers developed based on the relative differences in cost from the original Harding study (Table S5).

Table S6. Resource use multipliers per health states

| Health States | HS1 | HS2 | HS3 | HS4 | HS5 |
| --- | --- | --- | --- | --- | --- |
| Multiplier | 0.06 | 0.87 | 1 | 1.59 | 6.35 |

The dressing treatment mix and associated prices are depicted in Table S6

Table 7. Cost of dressings

| Dressing name: | Dressings per package | Size (cm) | Manufacturer | Price per pakage (PLN) |
| --- | --- | --- | --- | --- |
|  |  |  |  |  |
| Zetuvit Plus 10x10 cm, opatrunek z superabsorbentem, 100 cm2 | 1 | 10x10 | Paul Hartmann | 9,5 |
| Zetuvit Plus 10x20 cm, opatrunek z superabsorbentem, 200 cm2 | 1 | 10x20 | Paul Hartmann | 19,01 |
| Zetuvit Plus 20x40 cm, opatrunek z superabsorbentem, 800 cm2 | 1 | 20x40 | Paul Hartmann | 76,03 |
| Zetuvit Plus Silicone 10x20 cm, opatrunek z superabsorbentem i silikonową warstwą kontaktową, 200 cm² | 1 | 10x20 | Paul Hartmann | 19 |
| Zetuvit Plus Silicone 12,5x12,5 cm, opatrunek z superabsorbentem i silikonową warstwą kontaktową, 156.25 cm² | 1 | 12,5x12,5 | Paul Hartmann | 14,84 |
| Zetuvit Plus Silicone 20x25 cm, opatrunek z superabsorbentem i silikonową warstwą kontaktową, 500 cm² | 1 | 20x25 | Paul Hartmann | 47,5 |
| Zetuvit Plus Silicone Border 12,5x12,5 cm, samoprzylepny opatrunek z superabsorbentem i silikonową warstwą kontaktową, 156.25 cm² | 1 | 12,5x12,5 | Paul Hartmann | 14,84 |
| Zetuvit Plus Silicone Border 17,5x17,5 cm, samoprzylepny opatrunek z superabsorbentem i silikonową warstwą kontaktową, 306.25 cm² | 1 | 17,5x17,5 | Paul Hartmann | 29,08 |
| Zetuvit Plus Silicone Border 20x25 cm, samoprzylepny opatrunek z superabsorbentem i silikonową warstwą kontaktową, 500 cm² | 1 | 20x25 | Paul Hartmann | 47,5 |
| Biatain Silicone 12,5x12,5 cm, opatrunek piankowy z silikonem, 156.25 cm² | 1 | 12,5x12,5 | Coloplast | 14,85 |
| Allevyn Gentle Border 10x10 cm, opatrunek piankowy z warstwą adhezyjną zawierającą silikon, 100 cm² | 1 | 10x10 | S&N | 6,8 |
| Allevyn Gentle Border 10x10 cm, opatrunek piankowy z warstwą adhezyjną zawierającą silikon, 100 cm² | 1 | 10x10 | S&N | 6,8 |
| Allevyn Gentle Border 10x20 cm, opatrunek piankowy z warstwą adhezyjną zawierającą silikon, 200 cm² | 1 | 10x20 | S&N | 14,04 |
| Allevyn Gentle Border 10x20 cm, opatrunek piankowy z warstwą adhezyjną zawierającą silikon, 200 cm² | 1 | 10x20 | S&N | 14,04 |
| Allevyn Gentle Border 12,5x12,5 cm, opatrunek piankowy z warstwą adhezyjną zawierającą silikon, 156.25 cm² | 1 | Border | S&N | 10,69 |
| Allevyn Gentle Border 12,5x12,5 cm, opatrunek piankowy z warstwą adhezyjną zawierającą silikon, 156.25 cm² | 1 | Border | S&N | 10,69 |
| Allevyn Gentle Border 17,5x17,5 cm, opatrunek piankowy z warstwą adhezyjną zawierającą silikon, 306.25 cm² | 1 | Border | S&N | 21,49 |
| Allevyn Gentle Border 17,5x17,5 cm, opatrunek piankowy z warstwą adhezyjną zawierającą silikon, 306.25 cm² | 1 | Border | S&N | 21,49 |
| Allevyn Gentle Border 7,5x7,5 cm, opatrunek piankowy z warstwą adhezyjną zawierającą silikon, 56.25 cm² | 1 | Border | S&N | 4,07 |
| Allevyn Gentle Border 7,5x7,5 cm, opatrunek piankowy z warstwą adhezyjną zawierającą silikon, 56.25 cm² | 1 | Border | S&N | 4,07 |
| Allevyn Gentle Border Heel 23x23,2 cm, opatrunek piankowy z warstwą adhezyjną zawierającą silikon, 471 cm² | 1 | 23x23,2 | S&N | 33,48 |
| Allevyn Gentle Border Heel 23x23,2 cm, opatrunek piankowy z warstwą adhezyjną zawierającą silikon, 471 cm² | 1 | 23x23,2 | S&N | 33,48 |
| Allevyn Gentle Border Lite 10x10 cm, opatrunek piankowy z warstwą adhezyjną zawierającą silikon, 100 cm² | 1 | 10x10 | S&N | 6,8 |
| Allevyn Gentle Border Lite 10x10 cm, opatrunek piankowy z warstwą adhezyjną zawierającą silikon, 100 cm² | 1 | 10x10 | S&N | 6,8 |
| Allevyn Gentle Border Lite 15x15 cm, opatrunek piankowy z warstwą adhezyjną zawierającą silikon, 225 cm² | 1 | 15x15 | S&N | 16,2 |
| Allevyn Gentle Border Lite 15x15 cm, opatrunek piankowy z warstwą adhezyjną zawierającą silikon, 225 cm² | 1 | 15x15 | S&N | 16,2 |
| Allevyn Gentle Border Lite 8x8 cm, opatrunek piankowy z warstwą adhezyjną zawierającą silikon, 64 cm² | 1 | 8x8 | S&N | 4,63 |
| Allevyn Gentle Border Lite 8x8 cm, opatrunek piankowy z warstwą adhezyjną zawierającą silikon, 64 cm² | 1 | 8x8 | S&N | 4,63 |
| Allevyn Gentle Border Lite Oval 15,2x13,1 cm, opatrunek piankowy z warstwą adhezyjną zawierającą silikon, 156 cm² | 1 | 15,2x13,1 | S&N | 11,23 |
| Allevyn Gentle Border Lite Oval 15,2x13,1 cm, opatrunek piankowy z warstwą adhezyjną zawierającą silikon, 156 cm² | 1 | 15,2x13,1 | S&N | 11,23 |
| Allevyn Gentle Border Multisite 17,1x17,9 cm, opatrunek piankowy z warstwą adhezyjną zawierającą silikon, 225 cm² | 1 | Border | S&N | 15,66 |
| Allevyn Gentle Border Multisite 17,1x17,9 cm, opatrunek piankowy z warstwą adhezyjną zawierającą silikon, 225 cm² | 1 | Border | S&N | 15,66 |
| Allevyn Life 10,3x10,3 cm, opatrunek specjalistyczny - samoprzylepny, złożony, hydrokomórkowy opatrunek piankowy z żelem silikonowym o rozmiarze 10,3cm x 10,3cm, 93 cm² | 1 | 10,3x10,3 | S&N | 9,61 |
| Allevyn Life 10,3x10,3 cm, opatrunek specjalistyczny - samoprzylepny, złożony, hydrokomórkowy opatrunek piankowy z żelem silikonowym o rozmiarze 10,3cm x 10,3cm, 93 cm² | 1 | 10,3x10,3 | S&N | 9,61 |
| Allevyn Life 12,9x12,9 cm, opatrunek specjalistyczny - samoprzylepny, złożony, hydrokomórkowy opatrunek piankowy z żelem silikonowym o rozmiarze 12,9cm x 12,9cm, 146 cm² | 1 | 12,9x12,9 | S&N | 14,69 |
| Allevyn Life 12,9x12,9 cm, opatrunek specjalistyczny - samoprzylepny, złożony, hydrokomórkowy opatrunek piankowy z żelem silikonowym o rozmiarze 12,9cm x 12,9cm, 146 cm² | 1 | 12,9x12,9 | S&N | 14,69 |
| Allevyn Life 15,4x15,4 cm, opatrunek specjalistyczny - samoprzylepny, złożony, hydrokomórkowy opatrunek piankowy z żelem silikonowym o rozmiarze 15,4cm x 15,4cm, 211 cm² | 1 | 15,4x15,4 | S&N | 20,52 |
| Allevyn Life 15,4x15,4 cm, opatrunek specjalistyczny - samoprzylepny, złożony, hydrokomórkowy opatrunek piankowy z żelem silikonowym o rozmiarze 15,4cm x 15,4cm, 211 cm² | 1 | 15,4x15,4 | S&N | 20,52 |
| Allevyn Life 21x21 cm, opatrunek specjalistyczny - samoprzylepny, złożony, hydrokomórkowy opatrunek piankowy z żelem silikonowym o rozmiarze 21cm x 21cm, 392 cm² | 1 | 21x21 | S&N | 37,26 |
| Allevyn Life Heel 25x25,2 cm, opatrunek specjalistyczny - samoprzylepny, złożony, hydrokomórkowy opatrunek piankowy z żelem silikonowym o rozmiarze 25cm x 25,2cm kształt przystosowany do założenia na piętę, 545 cm² | 1 | 25x25,2 | S&N | 48,6 |
| Biatain Adhesive 10x10 cm, samoprzylepny opatrunek piankowy, 100 cm² | 1 | 10x10 | Coloplast | 9,93 |
| Biatain Adhesive 12,5x12,5 cm, samoprzylepny opatrunek piankowy, 156.25 cm² | 1 | 12,5x12,5 | Coloplast | 15,47 |
| Biatain Adhesive 18x18 cm, samoprzylepny opatrunek piankowy, 324 cm² | 1 | 18x18 | Coloplast | 32,12 |
| Biatain Adhesive 7,5x7,5 cm, samoprzylepny opatrunek piankowy, 56.25 cm² | 1 | 7,5x7,5 | Coloplast | 5,7 |
| Biatain Adhesive Heel 19x20 cm, samoprzylepny opatrunek piankowy na piętę, 380 cm² | 1 | 19x20 | Coloplast | 38,63 |
| Biatain Non Adhesive 10x10 cm, nieprzylepny opatrunek piankowy, 100 cm² | 1 | 10x10 | Coloplast | 11,19 |
| Biatain Non Adhesive 10x10 cm, nieprzylepny opatrunek piankowy, 100 cm² | 1 | 10x10 | Coloplast | 11,19 |
| Biatain Non Adhesive 15x15 cm, nieprzylepny opatrunek piankowy, 225 cm² | 1 | 15x15 | Coloplast | 24,12 |
| Biatain Non Adhesive 15x15 cm, nieprzylepny opatrunek piankowy, 225 cm² | 1 | 15x15 | Coloplast | 24,12 |
| Foam Lite Convatec opatrunek piankowy z silikonową warstwą kontaktową, przylepny 10x10 cm, opatrunek, 100 cm² | 1 | 10x10 | ConvaTec | 8,82 |
| Foam Lite Convatec opatrunek piankowy z silikonową warstwą kontaktową, przylepny 10x10 cm, opatrunek, 100 cm² | 1 | 10x10 | ConvaTec | 8,82 |
| Foam Lite Convatec opatrunek piankowy z silikonową warstwą kontaktową, przylepny 15x15 cm, opatrunek, 225 cm² | 1 | 15x15 | ConvaTec | 19,85 |
| Foam Lite Convatec opatrunek piankowy z silikonową warstwą kontaktową, przylepny 15x15 cm, opatrunek, 225 cm² | 1 | 15x15 | ConvaTec | 19,85 |
| Foam Lite Convatec opatrunek piankowy z silikonową warstwą kontaktową, przylepny 5,5x12 cm, opatrunek, 66 cm² | 1 | 5,5x12 | ConvaTec | 5,83 |
| Foam Lite Convatec opatrunek piankowy z silikonową warstwą kontaktową, przylepny 5,5x12 cm, opatrunek, 66 cm² | 1 | 5,5x12 | ConvaTec | 5,83 |
| Foam Lite Convatec opatrunek piankowy z silikonową warstwą kontaktową, przylepny 8x8 cm, opatrunek, 64 cm² | 1 | 8x8 | ConvaTec | 5,65 |
| Foam Lite Convatec opatrunek piankowy z silikonową warstwą kontaktową, przylepny 8x8 cm, opatrunek, 64 cm² | 1 | 8x8 | ConvaTec | 5,65 |
| Kliniderm Foam Border Silicone 10x10 cm, opatrunek, 100 cm² | 1 | 10x10 | Plastod | 9,4 |
| Kliniderm Foam Border Silicone 10x10 cm, opatrunek, 100 cm² | 1 | 10x10 | Plastod | 9,4 |
| Kliniderm Foam Border Silicone 15x15 cm, opatrunek, 225 cm² | 1 | 15x15 | Plastod | 21,15 |
| Kliniderm Foam Border Silicone 15x15 cm, opatrunek, 225 cm² | 1 | 15x15 | Plastod | 21,15 |
| Kliniderm Foam Border Silicone 15x20 cm, opatrunek, 300 cm² | 1 | 15x20 | Plastod | 28,19 |
| Kliniderm Foam Border Silicone 15x20 cm, opatrunek, 300 cm² | 1 | 15x20 | Plastod | 28,19 |
| Kliniderm Foam Border Silicone Heel 20x20,8 cm, opatrunek, 416 cm² | 1 | 20x20,8 | Plastod | 39,09 |
| Kliniderm Foam Border Silicone Heel 20x20,8 cm, opatrunek, 416 cm² | 1 | 20x20,8 | Plastod | 39,09 |
| Kliniderm Foam PHMB 10x10 cm, opatrunek, 100 cm² | 1 | 10x10 | Plastod | 9,4 |
| Kliniderm Foam PHMB 10x10 cm, opatrunek, 100 cm² | 1 | 10x10 | Plastod | 9,4 |
| Kliniderm Foam PHMB 15x15 cm, opatrunek, 225 cm² | 1 | 15x15 | Plastod | 21,15 |
| Kliniderm Foam PHMB 15x15 cm, opatrunek, 225 cm² | 1 | 15x15 | Plastod | 21,15 |
| Kliniderm Foam PHMB 20x20 cm, opatrunek, 400 cm² | 1 | 20x20 | Plastod | 37,58 |
| Kliniderm Foam PHMB 20x20 cm, opatrunek, 400 cm² | 1 | 20x20 | Plastod | 37,58 |
| Kliniderm Foam Silicone 10x10 cm, opatrunek, 100 cm² | 1 | 10x10 | Plastod | 9,4 |
| Kliniderm Foam Silicone 10x10 cm, opatrunek, 100 cm² | 1 | 10x10 | Plastod | 9,4 |
| Kliniderm Foam Silicone 15x15 cm, opatrunek, 225 cm² | 1 | 15x15 | Plastod | 21,15 |
| Kliniderm Foam Silicone 15x15 cm, opatrunek, 225 cm² | 1 | 15x15 | Plastod | 21,15 |
| Kliniderm Foam Silicone 20x20 cm, opatrunek, 400 cm² | 1 | 20x20 | Plastod | 37,58 |
| Kliniderm Foam Silicone 20x20 cm, opatrunek, 400 cm² | 1 | 20x20 | Plastod | 37,58 |
| Kliniderm Foam Silicone Heel 10x17,5 cm, opatrunek, 175 cm² | 1 | 10x17,5 | Plastod | 16,45 |
| Kliniderm Foam Silicone Heel 10x17,5 cm, opatrunek, 175 cm² | 1 | 10x17,5 | Plastod | 16,45 |
| Kliniderm Lite Foam Silicone 10x10 cm, opatrunek, 100 cm² | 1 | 10x10 | Plastod | 9,4 |
| Kliniderm Lite Foam Silicone 10x10 cm, opatrunek, 100 cm² | 1 | 10x10 | Plastod | 9,4 |
| Kliniderm Lite Foam Silicone 15x15 cm, opatrunek, 225 cm² | 1 | 15x15 | Plastod | 21,15 |
| Kliniderm Lite Foam Silicone 15x15 cm, opatrunek, 225 cm² | 1 | 15x15 | Plastod | 21,15 |
| Kliniderm Lite Foam Silicone 20X50 cm, opatrunek, 1000 cm² | 1 | 20X50 | Plastod | 93,96 |
| Kliniderm Lite Foam Silicone 20X50 cm, opatrunek, 1000 cm² | 1 | 20X50 | Plastod | 93,96 |
| Kliniderm Lite Foam Silicone 6x8,5 cm, opatrunek, 51 cm² | 1 | 6x8,5 | Plastod | 4,8 |
| Kliniderm Lite Foam Silicone 6x8,5 cm, opatrunek, 51 cm² | 1 | 6x8,5 | Plastod | 4,8 |
| Kliniderm Lite Foam Silicone Border 10x10 cm, opatrunek, 100 cm² | 1 | 10x10 | Plastod | 9,4 |
| Kliniderm Lite Foam Silicone Border 10x10 cm, opatrunek, 100 cm² | 1 | 10x10 | Plastod | 9,4 |
| Kliniderm Lite Foam Silicone Border 15x15 cm, opatrunek, 225 cm² | 1 | 15x15 | Plastod | 21,15 |
| Kliniderm Lite Foam Silicone Border 15x15 cm, opatrunek, 225 cm² | 1 | 15x15 | Plastod | 21,15 |
| Kliniderm Lite Foam Silicone Border 7,5x7,5 cm, opatrunek, 56.25 cm² | 1 | 7,5x7,5 | Plastod | 5,28 |
| Kliniderm Lite Foam Silicone Border 7,5x7,5 cm, opatrunek, 56.25 cm² | 1 | 7,5x7,5 | Plastod | 5,28 |
| Mepilex 10x21 cm, opatrunek, 210 cm² | 1 | 10x21 | Mölnlycke | 21,11 |
| Mepilex 10x21 cm, opatrunek, 210 cm² | 1 | 10x21 | Mölnlycke | 21,11 |
| Mepilex 12,5x12,5 cm, opatrunek, 156.25 cm² | 1 | 12,5x12,5 | Mölnlycke | 16,96 |
| Mepilex 12,5x12,5 cm, opatrunek, 156.25 cm² | 1 | 12,5x12,5 | Mölnlycke | 16,96 |
| Mepilex 17,5x17,5 cm, opatrunek, 306.25 cm² | 1 | 17,5x17,5 | Mölnlycke | 32,18 |
| Mepilex 17,5x17,5 cm, opatrunek, 306.25 cm² | 1 | 17,5x17,5 | Mölnlycke | 32,18 |
| Mepilex 20x50 cm, opatrunek, 1000 cm² | 1 | 20x50 | Mölnlycke | 105,08 |
| Mepilex 20x50 cm, opatrunek, 1000 cm² | 1 | 20x50 | Mölnlycke | 105,08 |
| Mepilex Border Flex Lite 10x10 cm, opatrunek, 100 cm² | 1 | 10x10 | Mölnlycke | 9,4 |
| Mepilex Border Flex Lite 10x10 cm, opatrunek, 100 cm² | 1 | 10x10 | Mölnlycke | 9,4 |
| Mepilex Border Flex Lite 15x15 cm, opatrunek, 225 cm² | 1 | 15x15 | Mölnlycke | 21,14 |
| Mepilex Border Flex Lite 15x15 cm, opatrunek, 225 cm² | 1 | 15x15 | Mölnlycke | 21,14 |
| Mepilex Border Flex Lite 7,5x7,5 cm, opatrunek, 56.25 cm² | 1 | 7,5x7,5 | Mölnlycke | 5,28 |
| Mepilex Border Flex Lite 7,5x7,5 cm, opatrunek, 56.25 cm² | 1 | 7,5x7,5 | Mölnlycke | 5,28 |
| Mepilex EM 12,5x12,5 cm, opatrunek, 156.25 cm² | 1 | 12,5x12,5 | Mölnlycke | 16,96 |
| Mepilex EM 12,5x12,5 cm, opatrunek, 156.25 cm² | 1 | 12,5x12,5 | Mölnlycke | 16,96 |
| Mepilex EM 17,5x17,5 cm, opatrunek, 306.25 cm² | 1 | 17,5x17,5 | Mölnlycke | 29,11 |
| Mepilex EM 17,5x17,5 cm, opatrunek, 306.25 cm² | 1 | 17,5x17,5 | Mölnlycke | 29,11 |
| Mepilex EM 7,5x8,5 cm, opatrunek, 63.75 cm² | 1 | 7,5x8,5 | Mölnlycke | 7,61 |
| Mepilex EM 7,5x8,5 cm, opatrunek, 63.75 cm² | 1 | 7,5x8,5 | Mölnlycke | 7,61 |
| Mepilex Talon 13X21 cm, opatrunek, 273 cm² | 1 | 13X21 | Mölnlycke | 21,98 |
| Mepilex Talon 13X21 cm, opatrunek, 273 cm² | 1 | 13X21 | Mölnlycke | 21,98 |
| Mepilex Transfer 15x20 cm, opatrunek jałowy, 300 cm² | 1 | 15x20 | Mölnlycke | 43,2 |
| Mepilex Transfer 15x20 cm, opatrunek jałowy, 300 cm² | 1 | 15x20 | Mölnlycke | 43,2 |
| Allevyn Adhesive 12,5x12,5 cm, opatrunek specjalistyczny - samoprzylepny, nawilżająca pianka poliuretanowa o rozmiarze 12,5cm x 12,5cm, 156.25 cm² | 1 | 12,5x12,5 | S&N | 7,08 |
| Allevyn Adhesive 12,5x12,5 cm, opatrunek specjalistyczny - samoprzylepny, nawilżająca pianka poliuretanowa o rozmiarze 12,5cm x 12,5cm, 156.25 cm² | 1 | 12,5x12,5 | S&N | 7,08 |
| Allevyn Adhesive 17,5x17,5 cm, opatrunek specjalistyczny - samoprzylepny, nawilżająca pianka poliuretanowa o rozmiarze 17,5cm x 17,5cm, 306.25 cm² | 1 | 17,5x17,5 | S&N | 13,89 |
| Allevyn Adhesive 17,5x17,5 cm, opatrunek specjalistyczny - samoprzylepny, nawilżająca pianka poliuretanowa o rozmiarze 17,5cm x 17,5cm, 306.25 cm² | 1 | 17,5x17,5 | S&N | 13,89 |
| Allevyn Adhesive 22,5x22,5 cm, opatrunek specjalistyczny - samoprzylepny, nawilżająca pianka poliuretanowa o rozmiarze 22,5cm x 22,5cm, 506.25 cm² | 1 | 22,5x22,5 | S&N | 21,92 |
| Allevyn Adhesive 22,5x22,5 cm, opatrunek specjalistyczny - samoprzylepny, nawilżająca pianka poliuretanowa o rozmiarze 22,5cm x 22,5cm, 506.25 cm² | 1 | 22,5x22,5 | S&N | 21,92 |
| Allevyn Heel 10,5x13,5 cm, opatrunek specjalistyczny - pianka poliuretanowa o rozmiarze 10,5cm x 13,5cm kształt przystosowany do założenia na piętę, 141.75 cm² | 1 | 10,5x13,5 | S&N | 7,57 |
| Allevyn Heel 10,5x13,5 cm, opatrunek specjalistyczny - pianka poliuretanowa o rozmiarze 10,5cm x 13,5cm kształt przystosowany do założenia na piętę, 141.75 cm² | 1 | 10,5x13,5 | S&N | 7,57 |
| Allevyn Non Adhesive 10x10 cm, opatrunek poliuretanowy, 100 cm² | 1 | 10x10 | S&N | 4,32 |
| Allevyn Non Adhesive 10x10 cm, opatrunek poliuretanowy, 100 cm² | 1 | 10x10 | S&N | 4,32 |
| Allevyn Non Adhesive 10x20 cm, opatrunek poliuretanowy, 200 cm² | 1 | 10x20 | S&N | 8,1 |
| Allevyn Non Adhesive 10x20 cm, opatrunek poliuretanowy, 200 cm² | 1 | 10x20 | S&N | 8,1 |
| Allevyn Non Adhesive 20x20 cm, opatrunek poliuretanowy, 400 cm² | 1 | 20x20 | S&N | 17,28 |
| Allevyn Non Adhesive 20x20 cm, opatrunek poliuretanowy, 400 cm² | 1 | 20x20 | S&N | 17,28 |
| Suprasorb P sensitive border 10x10 cm, opatrunek piankowy z silikonem, 100 cm² | 1 | 10x10 | Lohamnn&Rauscher | 9,02 |
| Suprasorb P sensitive border 12,5x12,5 cm, opatrunek piankowy z silikonem, 156.25 cm² | 1 | 12,5x12,5 | Lohamnn&Rauscher | 13,72 |
| Suprasorb P sensitive border 15x15 cm, opatrunek piankowy z silikonem, 225 cm² | 1 | 15x15 | Lohamnn&Rauscher | 20,2 |
| Suprasorb P sensitive border 20x20 cm, opatrunek piankowy z silikonem, 400 cm² | 1 | 20x20 | Lohamnn&Rauscher | 36,72 |
| Suprasorb P sensitive border 7,5x8,5 cm, opatrunek piankowy z silikonem, 63.75 cm² | 1 | 7,5x8,5 | Lohamnn&Rauscher | 5,92 |
| Suprasorb P sensitive border lite 10x10 cm, opatrunek piankowy z silikonem, 100 cm² | 1 | 10x10 | Lohamnn&Rauscher | 9,02 |
| Suprasorb P sensitive heel 25x23,5 cm, opatrunek piankowy z silikonem, 587.5 cm² | 1 | 25x23,5 | Lohamnn&Rauscher | 43,43 |
| Suprasorb P sensitive multisite 12x15 cm, opatrunek piankowy z silikonem, 180 cm² | 1 | 12x15 | Lohamnn&Rauscher | 15,55 |

Table 8. CHEERS 2022 Checklist

| Item # | CHEERS 2022 Item | Reported? | Where in Manuscript | Notes / Details |
| --- | --- | --- | --- | --- |
| 1 | Title – Identify study as an economic evaluation and specify interventions | Yes | Title | Cost-effectiveness stated; SAP vs Foam comparators explicitly named. |
| 2 | Abstract – Provide a structured summary of objectives, perspective, setting, methods, results, and conclusions | Yes | Abstract | Objectives, 6‑month horizon, Polish setting, results with costs, QALWs, healing; conclusion provided. |
| 3 | Background and objectives – Provide an explicit statement of the broader context and study objectives | Yes | Introduction; Aim | Prevalence/epidemiology and rationale; explicit aim to evaluate cost‑effectiveness/utility. |
| 4 | Target population and subgroups | Yes | Methods: Population and Data Sources | Adults with CLUs (venous/mixed), largely older population; no DFU included. |
| 5 | Settings and locations where data were collected | Yes | Introduction; Methods: Study Design and Setting | Polish healthcare system (NFZ), outpatient focus; context vs. other EU systems. |
| 6 | Study perspective | Yes | Methods: Study Design and Setting | Cost perspective of Polish healthcare system (NFZ). |
| 7 | Comparators | Yes | Methods: Treatment Modalities | Superabsorbent polymer (SAP; Zetuvit Silicone Border) vs foam dressings; both with standard care. |
| 8 | Time horizon | Yes | Abstract; Methods | Six months. |
| 9 | Discount rate | Yes | Methods: Methods for Cost Estimation | No discounting (time horizon < 1 year). |
| 10 | Choice of health outcomes | Yes | Methods: Effectiveness Evaluation | Healing rate and QALWs used (utilities 0–1; life tables for Poland). |
| 11 | Measurement of effectiveness | Yes | Methods; Supplementary (foam effectiveness SLR/meta) | SAP effectiveness from clinical studies; foam from SLR/meta; risk-prediction/transition model. |
| 12 | Measurement and valuation of preference-based outcomes | Yes | Methods: Effectiveness Evaluation; Table 3 | Utilities from literature (Clegg et al.); assumptions for some states; QALW aggregation. |
| 13 | Estimating resources and costs | Yes | Methods: Overview of Cost Components; Methods for Cost Estimation; Supplementary (resource tables) | Polish unit costs; health‑state costing framework; dressings cost handled separately; frequency of changes. |
| 14 | Rationale and data sources for resource use and cost | Yes | Methods; Supplementary | Harding method for health‑state multipliers; Polish price lists/tariffs; dressing price sources (2023). |
| 15 | Currency, price date, and conversion | Yes | Methods: Methods for Cost Estimation | PLN; adjusted for inflation/PPP where applicable; base year 2023. |
| 16 | Choice of model – Describe and justify model type and structure | Yes | Methods: Model Framework; Figure 2 | Individual‑level state‑transition (microsimulation) with weekly cycles; justified by heterogeneity. |
| 17 | Assumptions | Yes | Methods; Supplementary; Discussion | Equal transitions for some states; utility assumptions; standard care equal in both arms; early HTA framing. |
| 18 | Analytic methods (including handling of uncertainty, heterogeneity, and structural uncertainty) | Yes (uncertainty); Partial (heterogeneity) | Methods: Sensitivity Analyses; Supplementary | OWSA, PSA with distributions; heterogeneity beyond exudate class not analyzed; structural choices described. |
| 19 | Study parameters – Report values, ranges, references | Yes | Tables 1–4; Supplementary tables | Baseline characteristics, utilities, transitions, costs; SLR calibration factors for foams. |
| 20 | Incremental costs and outcomes | Yes | Results; Table 5 | SAP vs foam: −PLN 3,541; +0.123 QALWs; +2% healing; SAP dominates. |
| 21 | Characterizing uncertainty (e.g., PSA, OWSA) | Yes | Methods: Sensitivity Analyses; Results (Figures 3–4) | PSA cost‑saving in 100% simulations (reported); tornado for OWSA. |
| 22 | Characterizing heterogeneity | Partial / Not done | Discussion (limitations) | No subgroup CE by severity/classification; flagged as future research. |
| 23 | Distributional effects | Not reported | Discussion (limitations) | Authors note distributional effects not characterized due to data limitations. |
| 24 | Engagement with patients and others affected by the study | Partial | Discussion (limitations) | Clinicians involved; no patients/public/payers engaged; acknowledged as limitation. |
| 25 | Discussion of study findings, limitations, generalizability, and current knowledge | Yes | Discussion | Findings aligned with prior EU work; Polish context; limitations and implications discussed. |
| 26 | Conclusions and their implications | Yes | Conclusion | SAP cost‑saving and clinically favorable over 6 months; policy/practice implications noted. |
| 27 | Source of funding | Reported | After Conclusion Section | No founding. |
| 28 | Conflicts of interest and study registration | COI: reported / | After Conclusion Section | COI statement. |

**References**

1. Margolis DJ, Allen-Taylor L, Hoffstad O, Berlin JA. The accuracy of venous leg ulcer prognostic models in a wound care system. *Wound Repair Regen*. Mar-Apr 2004;12(2):163-8. doi:10.1111/j.1067-1927.2004.012207.x

2. Norman G, Westby MJ, Rithalia AD, Stubbs N, Soares MO, Dumville JC. Dressings and topical agents for treating venous leg ulcers. *Cochrane Database Syst Rev*. Jun 15 2018;6(6):Cd012583. doi:10.1002/14651858.CD012583.pub2

3. Guiding Principles for Monte Carlo Analysis (1997).

4. Clegg JP, Guest JF. Modelling the cost-utility of bio-electric stimulation therapy compared to standard care in the treatment of elderly patients with chronic non-healing wounds in the UK. *Curr Med Res Opin*. Apr 2007;23(4):871-83. doi:10.1185/030079906x167705

5. Matza LS, Kim KJ, Yu H, et al. Health state utilities associated with post-surgical Staphylococcus aureus infections. *Eur J Health Econ*. Aug 2019;20(6):819-827. doi:10.1007/s10198-019-01036-3

6. Vanscheidt W, Münter KC, Klövekorn W, Vin F, Gauthier JP, Ukat A. A prospective study on the use of a non-adhesive gelling foam dressing on exuding leg ulcers. *J Wound Care*. Jun 2007;16(6):261-5. doi:10.12968/jowc.2007.16.6.27063

7. Charles H, Callicot C, Mathurin D, Ballard K, Hart J. Randomised, comparative study of three primary dressings for the treatment of venous ulcers. *Br J Community Nurs*. Jun 2002;7(6 Suppl):48-54. doi:10.12968/bjcn.2002.7.Sup1.12965

8. Szewczyk MT, Mościcka P, Cwajda J, Piotrowicz R, Jawień A. Evaluation of the effectiveness of new polyurethane foam dressings in the treatment of heavily exudative venous ulcers. *Acta Angiologica*. 2007-04-17 2007;13(2):85-93.

9. Harding K, Posnett J, Vowden K. A new methodology for costing wound care. *Int Wound J*. Dec 2013;10(6):623-9. doi:10.1111/iwj.12006

10. Bartoszewicz M, Banasiewicz T, Bielecki K, et al. Zasady postępowania miejscowego i ogólnego w ranach/owrzodzeniach przewlekłych objętych procesem infekcji. *Forum Zakażeń*. 01/01 2019;10:1-30. doi:10.15374/FZ2019002

11. Waleria Hryniewicz JK, Tomasz Ozorowski, Anna Mól, Piotr Kulig, Dariusz Wąchol. *Stosowanie antybiotyków w wybranych zakażeniach skóry i tkanek miękkich*. Narodowy Instytut Leków; 2012.

12. Arkadiusz Jawień MTS, Andrzej Kaszuba, Zbigniew Gaciong, Zbigniew Krasiński, Jacek Wroński, Tomasz Grzela, Teresa Kobli. Guidelines for the management of chronic venous leg ulceration. Recommendations of a multidisciplinary expert group. 2011;8Leczenie Ran:59-80.
